# Pearl Oyster Bacterial Community Structure Is Governed by Location and Tissue-Type, but *Vibrio* Species Are Shared Among Oyster Tissues

**DOI:** 10.3389/fmicb.2021.723649

**Published:** 2021-08-09

**Authors:** William L. King, Mirjam Kaestli, Nachshon Siboni, Anna Padovan, Keith Christian, David Mills, Justin Seymour, Karen Gibb

**Affiliations:** ^1^Climate Change Cluster, University of Technology Sydney, Sydney, NSW, Australia; ^2^Research Institute for the Environment and Livelihoods, Charles Darwin University, Darwin, NT, Australia; ^3^Genecology Research Centre, University of the Sunshine Coast, Sunshine Coast, QLD, Australia

**Keywords:** pearl oyster (*Pinctada maxima*), *Vibrio*, bacterial communities, tissue-type, haemolymph, *hsp60*

## Abstract

Diseases of bivalves of aquacultural importance, including the valuable Australian silver-lipped pearl oyster (*Pinctada maxima*), have been increasing in frequency and severity. The bivalve microbiome is linked to health and disease dynamics, particularly in oysters, with putative pathogens within the *Vibrio* genus commonly implicated in oyster diseases. Previous studies have been biased toward the Pacific oyster because of its global dominance in oyster aquaculture, while much less is known about the microbiome of P. *maxima*. We sought to address this knowledge gap by characterizing the *P. maxima* bacterial community, and we hypothesized that bacterial community composition, and specifically the occurrence of *Vibrio*, will vary according to the sampled microenvironment. We also predicted that the inside shell swab bacterial composition could represent a source of microbial spillover biofilm into the solid pearl oyster tissues, thus providing a useful predictive sampling environment. We found that there was significant heterogeneity in bacterial composition between different pearl oyster tissues, which is consistent with patterns reported in other bivalve species and supports the hypothesis that each tissue type represents a unique microenvironment for bacterial colonization. We suggest that, based on the strong effect of tissue-type on the pearl oyster bacterial community, future studies should apply caution when attempting to compare microbial patterns from different locations, and when searching for disease agents. The lack of association with water at each farm also supported the unique nature of the microbial communities in oyster tissues. In contrast to the whole bacterial community, there was no significant difference in the *Vibrio* community among tissue types nor location. These results suggest that *Vibrio* species are shared among different pearl oyster tissues. In particular, the similarity between the haemolymph, inside shell and solid tissues, suggests that the haemolymph and inside shell environment is a source of microbial spillover into the oyster tissues, and a potentially useful tool for non-destructive routine disease testing and early warning surveillance. These data provide important foundational information for future studies identifying the factors that drive microbial assembly in a valuable aquaculture species.

## Introduction

There is growing evidence that the microbial communities living in association with a diverse range of animal hosts significantly contribute to host behavior, physiology and health (McFall-Ngai et al., 2013; Raina et al., 2018). Within a host, each tissue represents a unique microenvironment which facilitates distinct host-microbial interactions (Lokmer et al., 2016; King et al., 2020). For example, intestinal-associated microbial communities are commonly involved in nutrient mineralization and uptake for the host (Sonnenburg et al., 2004; Seth and Taga, 2014). For marine organisms, microbial communities contribute to important physiological processes including nutrient uptake and host defenses (Siboni et al., 2008; Glasl et al., 2016; Pita et al., 2018).

In recent years, diseases of marine organisms, particularly bivalves of aquacultural importance, have been increasing in frequency and severity (King et al., 2019a, b). There is a growing body of research that has linked microbiome composition to bivalve health and disease dynamics, particularly within oysters (Trabal et al., 2012; Trabal Fernández et al., 2014). For example, species assigned to the *Vibrio* genus are commonly implicated in oyster diseases (Wegner et al., 2013; Wendling et al., 2014; King et al., 2019a,b,c,d), whereby during the early stage of disease event, commensal vibrios are often replaced by phylogenetically similar pathogenic vibrios (Lemire et al., 2015). Therefore, it has been proposed that characterizing and understanding shifts in the *Vibrio* population could be important for predicting disease events (King et al., 2019c).

The silver-lipped pearl oyster (*Pinctada maxima*) is prized for its ability to produce large, high quality nacreous pearls and forms the basis of the valuable Australian pearling industry. At its peak, this industry was worth $200 million per year, yet is now valued at below $50 million per year, and still falling, due in part to largely unexplained disease events (Joint Select Committee on Northern Australia, 2016). Although there is an imperative to focus on pearl oyster health research to allow the industry to return pearl production as a major Australian Aquaculture industry, studies of bivalve bacterial composition (and the microbiome) are biased toward the Pacific oyster (*Crassostrea gigas*), because of its global dominance in oyster aquaculture (King et al., 2019b). While less is known about the microbiome of pearl oysters, Dubé et al. (2019) recently characterized the *P. margaritifera* microbiome using 16S rRNA gene sequencing and reported that the microbial communities were tissue specific.

*Vibrios* have been implicated in pearl oyster diseases internationally (Wang et al., 2016), including Australia (Negri et al., 2004), and were reported in low abundance in the *P. margaritifera* (Dubé et al., 2019) and *P. fucata martensii* bacterial communities (Zheng et al., 2021). It is still not clear what factors govern bacterial assemblage structure within the pearl oyster and to what extent putative pathogens, including *Vibrios*, influence pearl oyster health and productivity. Pearl oysters are subject to multiple stressors with the increasing adoption of aquaculture techniques (Adzigbli et al., 2020). Thus, to minimize the susceptibility of the oysters to disease, non-destructive, “least-stress” disease monitoring methods, such as inner shell swabs or haemolymph sampling, are needed. However, these approaches would only be of value if the microbiome of these samples were representative of those from other tissues.

Given the importance of microbial communities in physiological processes and disease prediction, our first objective was to provide the first characterization of the bacterial community of *P. maxima* by defining the bacterial communities in different oyster tissues. A second objective was to determine patterns in *Vibrio*, given their implication in disease in other oyster species. Based on previous studies, we hypothesized that bacterial and *Vibrio* composition would vary according to the sampled microenvironment and that the inside shell swab bacterial composition could represent a source of microbial spillover biofilm into the solid pearl oyster tissues, thus providing a useful predictive sampling environment. In addition to providing evidence in support of a non-destructive sampling technique, this study provides foundational information about the microbial assembly in a valuable, understudied aquaculture species.

## Meterials and Methods

### Sites and Sample Processing

*Pinctada maxima* tissue and seawater samples were collected in April 2018 from two pearl oyster farm sites in Western Australia, namely Seaflower Bay (SF) and Wargul Wargul Bay (WW), which are located in the Vansittart Bay region (12.438241 S 130.796684 E) of the northern Kimberley. Three unseeded oysters ranging in size from 90 to 95 mm (dorso-ventral) were harvested from SF (numbers limited as they were brood stock) and 10 oysters (seeded 22 months earlier) ranging from 150 to 170 mm (dorso-ventral) were harvested from WW. The outer shell of each oyster was swabbed using sterile Copan Rayon Tip swabs (Interpath Services, catalog:155CIS). A processing knife was used to cleanly sever the adductor muscle of each oyster, before the nacreous inside shell surface was swabbed. The mantle was then detached from one shell valve using a sterile scalpel blade, and using a 22-gauge needle, 200–500 μL of haemolymph was collected from the auricle. Tissue from the mantle, gill, digestive diverticula, large intestine and heart (auricles and ventricle) were dissected from each oyster and all samples were placed into separate, sterile tubes and stored at −80°C until extraction. Seawater (250 mL) collected from each site was filtered through a 0.22 μm mixed cellulose membrane.

Genomic DNA (gDNA) from the tissue and haemolymph samples was extracted using the Qiagen DNeasy Blood and Tissue Kit (catalog: 69504) according to the manufacturer’s instructions. The swabs and filtered water samples were extracted using the MP Biomedicals FastDNA^TM^ SPIN Kit for Soil (catalog: 6560200). Purity ratio (260/280) and the DNA concentration in each sample was quantified using the NanoDrop^TM^ One (Thermo Scientific^TM^). A total of 106 samples were processed across the two oyster farms.

### 16S rRNA and *Vibrio*-Centric *hsp60* Amplicon Sequencing

DNA was amplified using: (1) the primers Bakt_341F and Bakt_805R which amplify the V3-V4 region of the bacterial 16S rRNA gene (Herlemann et al., 2011) and (2) the *Vibrio*-centric *hsp60* primers Vib-hspF3-23 and Vib-hspR401-422, as previously described (King et al., 2019c), to characterize the composition and diversity of the entire bacterial assemblage and *Vibrio* community, respectively. PCR conditions for 16S rRNA amplification were as follows: 95°C for 3 min, 25 cycles of 95°C for 30 s, 55°C for 30 s, and 72°C for 30 s, and a final extension at 72°C for 5 min. For the *Vibrio*-centric *hsp60* assay a 30 μL PCR reaction mixture was prepared using an epMotion 5075l Automated Liquid Handling System (Eppendorf South Pacific) to limit cross sample contamination and the PCR protocol was performed as previously described (King et al., 2019c). Amplicons were sequenced using the Illumina MiSeq platform according to the manufacturer’s guidelines (Ramaciotti Centre for Genomics, Sydney, NSW, Australia). Raw data files in FASTQ format were deposited in NCBI Sequence Read Archive (SRA) under Bioproject number PRJNA594420.

### *Vibrio*-Centric Quantitative PCR (qPCR)

To provide a measure of *Vibrio* abundance, a quantitative PCR (qPCR) assay was used to quantify *Vibrio*- specific 16S rRNA gene copies in each sample as previously described (Thompson et al., 2004; Siboni et al., 2016; King et al., 2019c). The resulting data were normalized to milliliters of collected water or milligrams of tissue. Swabs of out- and inside of shells were excluded from this analysis.

### Sequence Processing

16S rRNA gene amplicon raw demultiplexed data was processed using the Quantitative Insights into Microbial Ecology (QIIME 2 version 2018.6.0) pipeline (Bolyen et al., 2019). Briefly, paired-ended sequences were imported using the “qiime tools import” command. Sequences were then trimmed and denoised using DADA2 version 1.6, which also removes chimeras (Callahan et al., 2016). Taxonomy was assigned on the rep-set-dada2 output at the single nucleotide level using the sklearn qiime feature classifier against the Silva v132 database (Quast et al., 2013). Sequences identified at the single nucleotide threshold are henceforth denoted as amplicon sequence variants (ASVs). The dataset was further cleaned by removing ASVs which only occurred in one sample and those identified as non-bacterial, chloroplasts or mitochondria. Cleaned data were rarefied at 1,220 reads per sample with 63 samples remaining and 1,263 ASVs. Rarefaction curves indicated that the ASV richness plateaued for the majority of samples at this depth with the exception of outside shell samples.

For the *Vibrio*-centric *hsp60* data, raw pair-ended sequences were joined using FLASH (Magoč and Salzberg, 2011) and trimmed using Mothur (Schloss et al., 2009) (parameters: maxhomop = 5, maxambig = 0, qaverage = 25, minlength = 420, maxlength = 420). These fragments were then clustered at 97% into OTUs and chimeric sequences were removed using vsearch (Rognes et al., 2016). Non-*Vibrio* sequences were removed by BLASTing cleaned sequences against the *Vibrio hsp60* reference dataset and any sequence with similarity lower than 90% was removed. This fasta file was then used to assign taxonomy against the custom *Vibrio hsp60* reference dataset with the RDP classifier. OTUs that only occurred in one sample were excluded and samples with less than 10 *hsp60* sequences were also excluded. This resulted in 41 samples and 24 *hsp60* OTUs. Due to the large spread of sequences per sample (11–29,163), data were not rarefied, rather sequences were normalized to the number of sequences per sample to produce the relative abundance of each taxa for each sample.

### Data Analysis

All analyses were conducted in R v4.0.2 and Primer-E (v7, Quest Research Limited). Bacterial richness (observed number of ASVs based on rarefied sequencing data) was compared between locations (# 2) and tissue types (# 8 shell and tissues types) plus farm water using a quasi-Poisson model with sample type (oyster tissue type and water) and location as fixed factors (estimated overdispersion parameter 22). Model residuals were checked for influential outliers and lack of patterns across predictors and fitted values. Oysters (# 12) were added as random intercept in a mixed effect negative binomial model (glmmTMB package – nbinom1 family), but no between-oyster variance in the richness was found and the quasi-Poisson GLM model was used instead.

To visualize differences between bacterial compositions for different oyster tissue types, a Principal Coordinates Analysis (PCoA) was performed in the Phyloseq package on the Bray-Curtis dissimilarity matrix of the square-root transformed and rarefied ASV data (McMurdie and Holmes, 2013). Similarly, in order to examine whether the bacterial compositions differed between tissue types and location, a PERMANOVA test was conducted in Primer-E on the dissimilarity matrix with 999 permutations, type III sums of squares and oysters as random effect nested in location.

To identify taxa that were associated with specific oyster tissue types, a Dufrene-Legendre Indicator Species Analysis (IndVal) was performed (labdsv package) (De Cáceres et al., 2010). *P*-values were adjusted for multiple testing using the FDR method. *Vibrio* abundance based on *Vibrio* 16S rRNA gene qPCR data normalized per mg tissue or mL water (shell swabs excluded) was compared between tissue types and location using a Gamma (log link) mixed effect model with oysters as random intercept (glmmTMB package). No between-oyster variance in the *Vibrio* abundance was found and the random intercept was dropped. A PERMANOVA analysis on the *Vibrio hsp60* composition was conducted as per above, whereby the relative abundances of *hsp60* sequences were used in the absence of rarefied data.

## Results

### Bacterial Richness Was Greatest for the Outside Shell Microbiota

**FIGURE 1:**
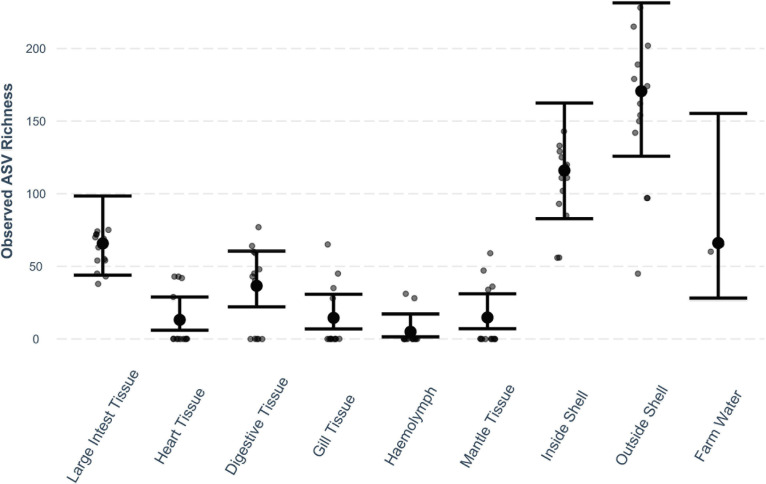
Observed ASV richness (small dots) across oyster tissue types and farm water. Mean estimates (large dots) are based on a quasi-Poisson model with fixed factors oyster tissue type and location. Error bars mark 95% confidence intervals.

The observed bacterial richness significantly differed between oyster tissue types (Figure 1). Richness was significantly higher in the outer shell than in all other tissue types (*P* < 0.001 for all) and the inside shell and farm water (*P* < 0.05 for both). Accounting for oyster tissue type, there was no difference in bacterial community richness between locations.

### Bacterial Composition Differed According to Location and Pearl Oyster Tissue Type

**FIGURE 2:**
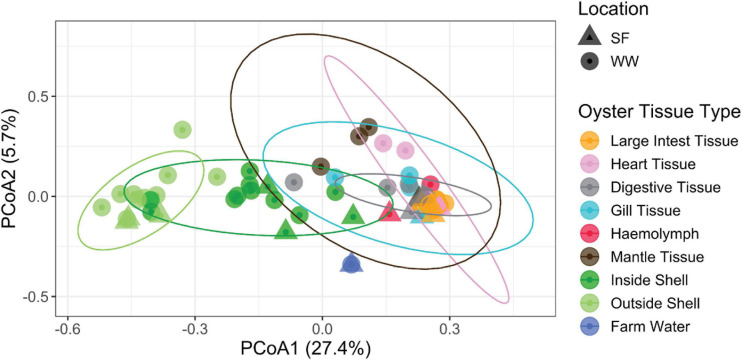
PCoA ordination of bacterial compositions labeled by location and oyster tissue type. The first axis explained 27.4% of the microbiota variance and the second 5.7%. Ellipses show 95% data distribution.

**FIGURE 3:**
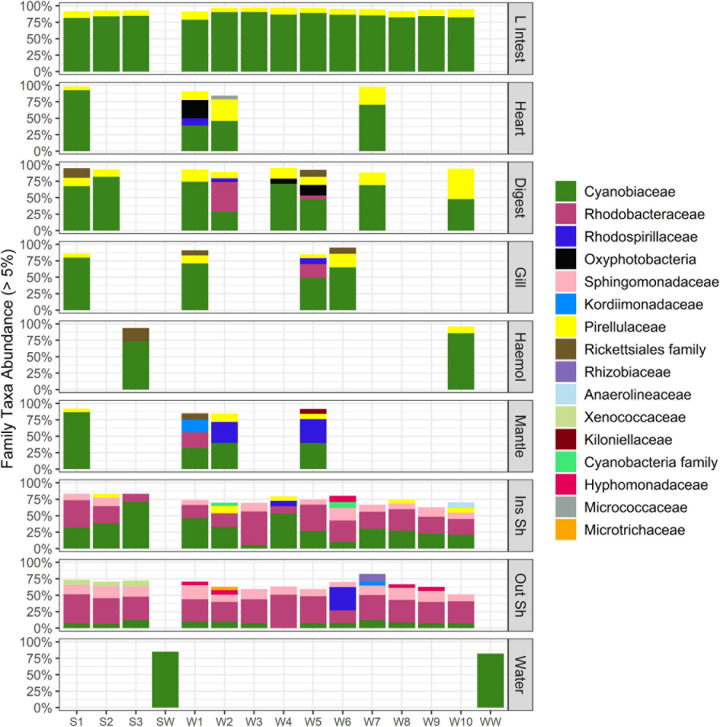
Stacked bar plot of bacterial taxa at family level (relative abundance > 5%) across oyster tissue types and farm water. Each column is a sample. “L Intest” large intestinal tissue, “Digest” digestive tissue, “Haemol” haemolymph, “Ins Sh” inside shell, “Out Sh” outside shell, “Water” farm water. “S,” Seaflower Bay and “W,” Wargul Wargul Bay. Only samples with > 1220 sequence reads were included.

Pearl oyster-associated bacterial communities differed according to farm location (PERMANOVA: Pseudo *F*_1_, _34_ = 2.0, *P* = 0.001) and oyster tissue (Pseudo *F*_7_, _34_ = 3.0, *P* = 0.001) (Figure 2). In particular, bacterial communities on the outside shell differed from most other tissues including the inside shell, while bacterial assemblages of the latter also differed from the digestive tissue (*P* < 0.01 for all). The bacterial communities within the large intestinal tissue showed the lowest variability displaying a similar community structure across all replicates and both locations (Figure 2) at family (Figure 3) and genus (Supplementary Tables 1, 2) levels. A triangle heat map of average inter-group Bray-Curtis similarities between tissue types illustrates that bacterial communities on the outside shell differed from most other tissues including the inside shell, and the bacterial communities within the large intestinal tissue had the most similar community structure across all replicates and both locations (Supplementary Figure 1).

*Cyanobiaceae* were the most dominant family across all oyster tissue types and farm water, with average relative abundance of 49%. The second and third most dominant families were *Rhodobacteraceae* and *Pirellulaceae* representing 15 and 8.5% average relative abundance, respectively (Figure 3). The dominance of the *Cyanobiaceae* family largely contributed to its over-representation in the oyster tissues (average relative abundance 54%) compared to the inside and outside of the shell (15%) and was largely *Synechococcus* CC9902 (Supplementary Tables 1, 2). The *Pirellulaceae* (Planctomycete) were represented by one genus, *Blastopirellula* (Supplementary Tables 1, 2). *Rhodobacteraceae* were over-represented in the inside and outside of the shell (24%) relative to the grouped oyster tissues (4%) comprising the genera *Ruegeria, Nautella, Silicimonas*, and *Actibacterium* (Supplementary Tables 1, 2).

Indicator Species Analysis was used to identify bacterial families that were associated with a sampled environment (Table 1). The outside shell bacterial composition had the greatest number of taxa which were significantly associated with a single oyster tissue type (9 families). These families occurred in 77–100% of outside shell samples (*n* = 13) and 41–81% of their occurrence in the dataset was in these samples. In particular, *Xenococcaceae* and *Synechococcales* almost exclusively occurred in the outer shell (80% of counts with remaining counts mainly occurring in the inside shell) and more than 90% of outer shell samples contained these taxa. One family each was significantly associated with the inside shell (an uncultured Cyanobacterial family) and the large intestinal tissue (a Planctomycetales family). Seven Alpha- and Deltaproteobacterial taxa were associated with the farm water samples; these were mainly marine SAR11, 116, and 324 clade families.

**TABLE 1:** Indicator species analysis showing bacterial taxa at family level which were significantly associated with different oyster tissue types or farm water.

**Sample type**	**Taxa**	***P*-value (IndVal)**
Farm water	SAR11 clade family	0.012 (1.0)
	SAR324 cluster family	0.012 (1.0)
	SAR116 clade family	0.012 (1.0)
	SAR324 clade family	0.014 (1.0)
	*Rhodospirillales AEGEAN-169 marine group*	0.006 (0.98)
	SAR11 clade family	0.006 (0.98)
	*Actinomarinaceae*	0.005 (0.94)
Outside shell	Synechococcales family	0.002 (0.75)
	*Xenococcaceae*	0.002 (0.74)
	Rhizobiales family	0.002 (0.65)
	*Hyphomonadaceae*	0.002 (0.63)
	*Parvularculaceae*	0.050 (0.62)
	*Sphingomonadaceae*	0.002 (0.52)
	*Methyloligellaceae*	0.042 (0.49)
	*Hyphomicrobiaceae*	0.019 (0.46)
	*Rhodobacteraceae*	0.002 (0.41)
Inside shell	Cyanobacterial family	0.021 (0.71)
Large intestinal tissue	Planctomycetales family	0.050 (0.58)

*P-values are FDR corrected for multiple testing. The test statistic IndVal is in brackets.*

### *Vibrio* Abundance Differed According to Pearl Oyster Tissue Type

*Vibrio* abundance significantly differed according to oyster tissue type (*P* < 0.001), but did not differ between locations (*P* = 0.4). *Vibrio* abundance in the large intestine tissue was greater than in all other tissue types (Tukey adjusted *P* < 0.01 for all except *P* = 0.013 for digestive tissue and *P* = 0.057 for water). More specifically, it was on average 32 times greater than the digestive tissue, 44 and 80 times for gill and haemolymph tissue and 280 and 600 times greater than the heart and mantle tissue.

### *Vibrio* Community Patterns Did Not Differ According to Pearl Oyster Tissue Type

**FIGURE 4:**
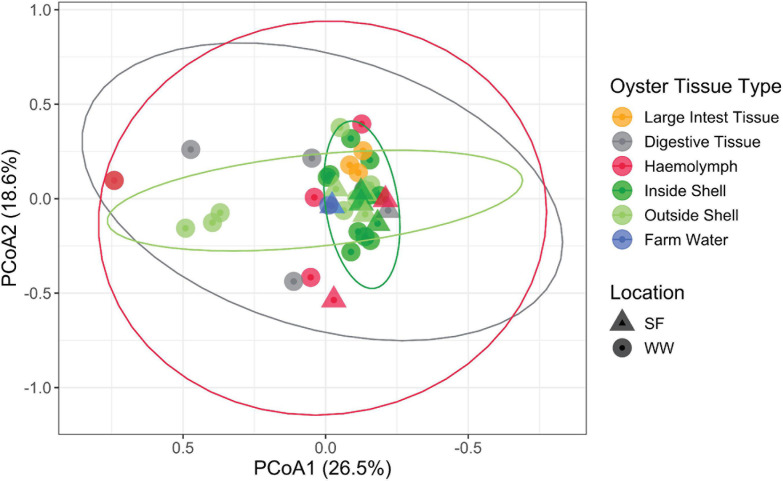
PCoA ordination of the *Vibrio* compositions based on hsp60 sequences labeled by location and oyster tissue type. The first axis explained 26.5% of the *Vibrio* community variance and the second 18.6%. Ellipses show 95% data distribution.

**FIGURE 5:**
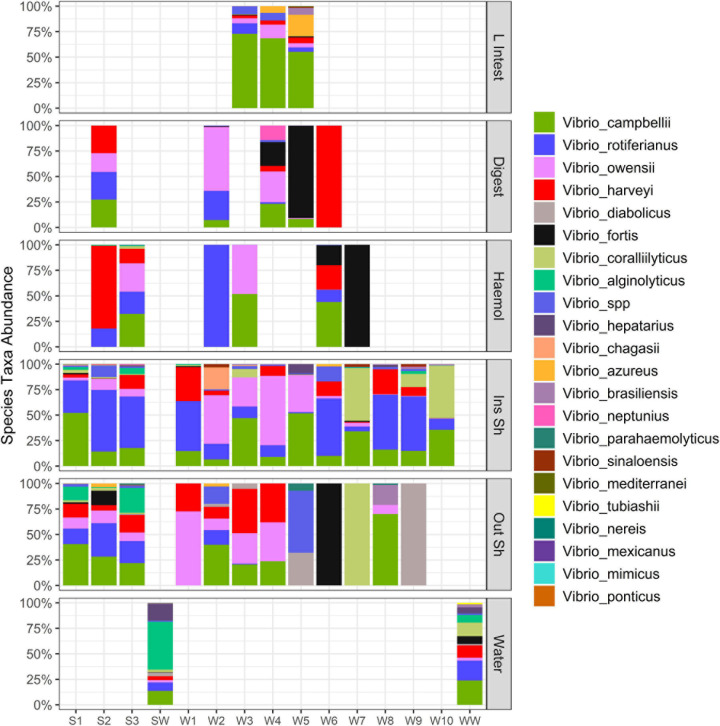
Stacked bar plot of *Vibrio* species across oyster tissue types and farm water. Each column is a sample. “L Intest” large intestinal tissue, “Digest” digestive tissue, “Haemol” haemolymph, “Ins Sh” inside shell, “Out Sh” outside shell, “Water” farm water. “S,” Seaflower Bay and “W,” Wargul Wargul Bay. Only samples with > 10 sequence reads were included.

Due to the significantly elevated *Vibrio* abundance in the various tissue types, we sought to identify the species present and discern patterns in the *Vibrio* community using the *hsp60* gene as a taxonomic marker. By using this marker gene and after cleaning the dataset, 41 samples were characterized. The majority of these samples were from the inside (*n* = 13) and outside shell (*n* = 12), followed by the haemolymph (*n* = 6), digestive tissue (*n* = 5), large intestinal tissue (*n* = 3) and farm water (*n* = 2). Notably, no *hsp60* sequences were recovered from the heart, gill nor mantle tissue. In contrast to the whole bacterial community, there was no specific *Vibrio* fingerprint or clustering of *Vibrio* communities by oyster tissue type with the exception of the large intestinal tissue (Figures 4, 5). A triangle heat map of average inter-group Bray-Curtis similarities between tissue types confirmed that with the exception of the large intestinal tissue the levels of similarity were generally evenly distributed within and between tissues types indicating no specific *Vibrio* fingerprint for a specific tissue type (Supplementary Figure 2).

Of the 24 unique *Vibrio* species detected, *Vibrio campbellii*, *Vibrio rotiferianus, Vibrio owensii*, and *Vibrio harveyi* were the most dominant members recovered from oysters, representing an average relative abundance of 25, 18, 16, and 14%, respectively (Figure 5). Indicator species analysis identified *V*. *hepatarius, V. diabolicus, and V. tubiashii* were associated with farm water (IndVal > 0.75, FDR adjusted *P*-value < 0.05), however in keeping with the observed high variability of the *Vibrio* community within oyster tissue types (Figures 4, 5), no *Vibrio* species was significantly associated with a specific oyster tissue.

## Discussion

Here we provide the first characterization of the bacterial community associated with the silver-lipped pearl oyster (*Pinctada maxima*). This study revealed that, as has been observed in other oyster species (King et al., 2020), discrete bacterial communities are associated with different pearl oyster tissue types. This finding supports the hypothesis that each tissue type represents a unique microenvironment for bacterial colonization and offers differences in niche space available for bacterial colonization (Dubé et al., 2019; Pathirana et al., 2019; King et al., 2020). The lack of association with water at each farm also supports the unique nature of the microbial communities in oyster tissues. The dominance of the *Cyanobiaceae* genus *Synechococcus* in both oyster tissue type and farm water has been reported elsewhere including in the pearl oyster intestine and surrounding water environment (Zheng et al., 2021). The higher relative abundance of the *Rhodobacteraceae* family in the shell swab samples relative to the pearl oyster tissues samples suggests the preferential colonization of these species on the shell. This family included the genera *Nautella* and *Ruegeria* reported in other pearl oyster species (Zheng et al., 2021) and members of the genus *Ruegeria* can produce the broad-spectrum antibiotic tropodithietic acid (Beyersmann et al., 2017). The dominance in the shell microbiome supports their reported ability to rapidly colonize surfaces and produce antibacterial components, preventing other bacteria from growing (Arfken et al., 2017). *Ruegeria* spp. have been used in aquaculture to suppress growth of marine pathogens including *Vibrio* sp., and have potential as probiotic or antifouling agents (Berger et al., 2011). Notably, some genera within the *Rhodobacteraceae* family are also known for their ability to metabolize calcium compounds and could be using the shell as a source of nutrients (Pujalte et al., 2014).

The pearl oyster large intestinal tissue was remarkably different from all other tested tissue types and was dominated by an uncultured family in the *Planctomycetales*. In agreement with our data, *Planctomycete* bacteria have been identified as dominant members within the intestinal and gut environment in many bivalve species including pearl oysters (Dubé et al., 2019; Zheng et al., 2021). In our study, the dominant genus *Blastopirellula* is in the family *Pirellulaceae*, members of which are dominant in pearl oyster alimentary tissue (Dubé et al., 2019). Members of this family can reportedly exploit sulfated algal polysaccharides that might be commonly ingested by oysters as a consequence of phytoplankton consumption. All *Blastopirellula* characterized to date use a wide range of simple non-sulfated sugars, at least some of which are likely to occur in the oyster digestive tract as algal biomass is hydrolyzed (King et al., 2012). Because of the low between-replicate variability and species evenness in the intestinal tissue, it suggests that this tissue microenvironment is relatively stable and could be a suitable environment for bacteria with long generation times.

When we measured tissue specificity for the *Vibrio* community using the *hsp60 Vibrio* taxonomic marker we found that for those tissues with *hsp60* genes detected, unlike the whole bacterial community analysis, there was no tissue specificity, with the exception of the large intestine. *V*. *campbellii* was the dominant member across all samples, primarily driven by its over-representation in the intestinal tissue. *V. campbellii* has been implicated in shrimp diseases (Haldar et al., 2011; Vicente et al., 2020), but little is known about its associations with oysters. In addition, *V. rotiferianus, V. owensii* and *V. harveyi* were the most dominant members recovered from oysters and these were present in outer and inner shell swabs, haemolymph, digestive tissue, large intestine and seawater, but not heart, gill nor mantle tissue. These three species have been isolated from moribund Pacific oysters (Wang et al., 2021) and while *V. harveyi* is a known oyster pathogen, implicated in Pacific oyster mortality events (King et al., 2019c), less is known about *V. rotiferianus* and *V. owensii.* Wang et al. (2021) demonstrated that *V. owensii* had low pathogenicity in Pacific oysters, however, this species is implicated in an emergent shrimp disease (Liu et al., 2021). *Vibrio fortis* was dominant in some samples and interestingly this species and *V. harveyi* increased dramatically in simulated heat wave experiments associated with Pacific oyster mortality, which implicates them as pathogens, cooperatively or independently (Green et al., 2019).

There is a paucity of studies characterizing whole *Vibrio* community using culture-independent techniques in bivalves and we are not aware of studies that have described multiple *Vibrio* species distribution in pearl oyster tissue because the 16S rRNA approach generally does not resolve vibrios (Dubé et al., 2019). Our finding that vibrios colonize a wide variety of pearl oyster tissues, including the shell, may reflect the fact that oysters represent a potentially important ecological niche for these bacteria. Furthermore, vibrios are known for their expansive metabolic capabilities and are able to colonize a multitude of environments (Thompson et al., 2004; Grimes et al., 2009). Consequently, these vibrio communities are potentially not host specific and rather reflect a random assemblage of *Vibrio* spp. influenced by the surrounding environment (Wendling et al., 2014; Wendling and Wegner, 2015). It may also be the case that the *Vibrio* community, including those in the haemolymph can persist in the oyster tissues due to a lack of sensitivity to the bactericidal activity of the haemolymph (Pruzzo et al., 2005).

It has been suggested that the oyster haemolymph provides a good indication of overall oyster health and is where disease is likely to manifest (Lokmer and Wegner, 2015; King et al., 2019b). Shifts in the oyster haemolymph microbiome have been linked to disease (Lemire et al., 2015; Lokmer and Wegner, 2015). Routine haemolymph sampling could be an early detection, disease prevention tool. For example, Lokmer et al. (2016) reported a spillover of cultivable Vibrionaceae from the haemolymph into solid tissues during a disease event. Furthermore, we have demonstrated that several known and potential pathogenic *Vibrio* spp. were detected in *P. maxima* haemolymph which supports a capacity for routine and early detection in the event of disease symptoms.

The similarity between the inside shell and pearl oyster tissues vibrio compositions could suggest that the inside shell environment is also a source of microbial spillover into the oyster tissues. In support of this observation, a bacterial pathogen of the Pacific oyster preferentially colonizes the inner shell and causes tissue pathology from this microenvironment (Boardman et al., 2008). This may also be relevant for non-vibrio pathogens. For example, studies of a bacterial pathogen (*Roseovarius crassostreae*) of the cultured eastern oysters (*Crassostrea virginica*) indicate colonization on the inside shell microenvironment and movement and pathology on solid oyster tissues (Boardman et al., 2008), suggesting that microbial spillover from the inside-shell microbial biofilm into the oyster is plausible. Understanding the links between the shell and oyster tissue bacterial community is important, because analysis of the shell bacterial community may provide opportunities for non-invasive sampling of the oyster bacterial community *in situ*. Our results show that the inner shell swab also has potential as a less stressful routine surveillance tool and is easily sampled when the shell is being subjected to routine farming techniques.

## Conclusion

In recent years, interest in using the bivalve microbiome to detangle disease dynamics has exponentially increased. However, it is necessary to build a foundation of the factors that govern bivalve microbiome assembly to understand how the bivalve microbiome could contribute to disease processes. Our bacterial community diversity study has addressed a knowledge gap for the commercially important pearl oyster, *P. maxima*, whereby we have shown that pearl oyster bacterial composition is governed by both location and tissue-type, which is consistent with observations in other bivalve species. Interestingly, there was no significant difference in the *Vibrio* community between tissue types nor location. These results imply that *Vibrio* species are shared among different pearl oyster tissues. In particular, the similarity between the haemolymph, inside shell and solid tissues suggests that they are a source of microbial spillover into the oyster tissues, and a potentially useful tool for non-destructive routine disease testing and early warning surveillance. Based on the strong effect of tissue-type on the pearl oyster bacterial community, future studies should apply caution when attempting to compare microbial patterns with current literature, particularly from different locations and microenvironments, and when searching for disease agents. The bacterial community analyses and conclusions are based on 63 samples and 1,263 ASVs (following rarefaction), and while larger sample sets could be analyzed in the future, this work provides important foundational information for future studies identifying the factors that drive microbial assembly in a valuable aquaculture species.

## Data Availability Statement

The raw data files in FASTQ format were deposited in NCBI Sequence Read Archive (SRA) under BioProject ID: PRJNA594420. Data analysis workflow, reference data set and taxonomy file are available at https://doi.org/10.17605/OSF.IO/4798P.

## Author Contributions

WK and NS created the degenerate primers and optimized the PCR. MK and WK analyzed the data. DM provided samples. AP was responsible for laboratory work. KG, AP, DM, and KC conceived and designed the study. KG, WK, MK, AP, KC, and JS wrote the manuscript. All authors contributed to the article and approved the submitted version.

## Conflict of Interest

The authors declare that the research was conducted in the absence of any commercial or financial relationships that could be construed as a potential conflict of interest.

## Publisher’s Note

All claims expressed in this article are solely those of the authors and do not necessarily represent those of their affiliated organizations, or those of the publisher, the editors and the reviewers. Any product that may be evaluated in this article, or claim that may be made by its manufacturer, is not guaranteed or endorsed by the publisher.

## Supplementary Material

Supplementary Figure 1 Heat map triangle showing average inter-group Bray-Curtis similarities of the bacterial communities between tissue types. The circles show the average within-tissue type similarities and larger circles and numbers represent greater similarities within and between tissue types.

Supplementary Figure 2 Heat map triangle showing average inter-group Bray-Curtis similarities of the *Vibrio* communities between tissue types. The circles show the within-tissue type similarities and larger circles and numbers represent greater similarities within and between tissue types.

Supplementary Table 1 Relative abundance (>1%) of bacterial taxa to genus level associated with different oyster tissue types.

Supplementary Table 2 Relative abundance (>1%) of bacterial taxa to genus level associated with different oyster tissue types grouped by location.
