# Supplementary figures and images for: Pearl Oyster Bacterial Community Structure Is Governed by Location and Tissue-Type, but Vibrio Species Are Shared Among Oyster Tissues
Source: Front Microbiol. 2021 Aug 9;12:723649. doi: 10.3389/fmicb.2021.723649 (PMC8381468; doi:10.3389/fmicb.2021.723649)

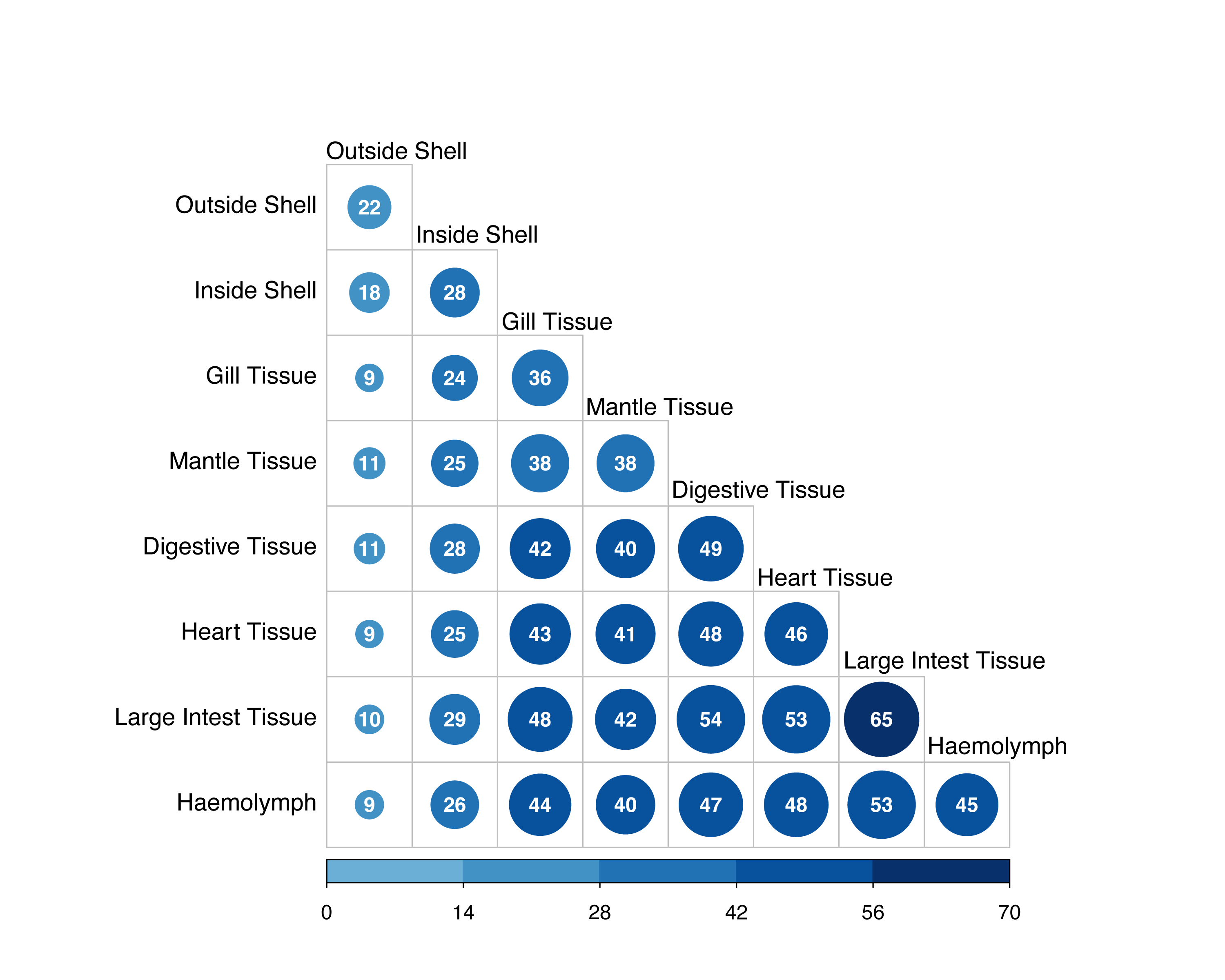

Supplement: Supplementary Figure 1 — Heat map triangle showing average inter-group Bray-Curtis similarities of the bacterial communities between tissue types. The circles show the average within-tissue type similarities and larger circles and numbers represent greater similarities within and between tissue types. [file Image_1.TIFF]

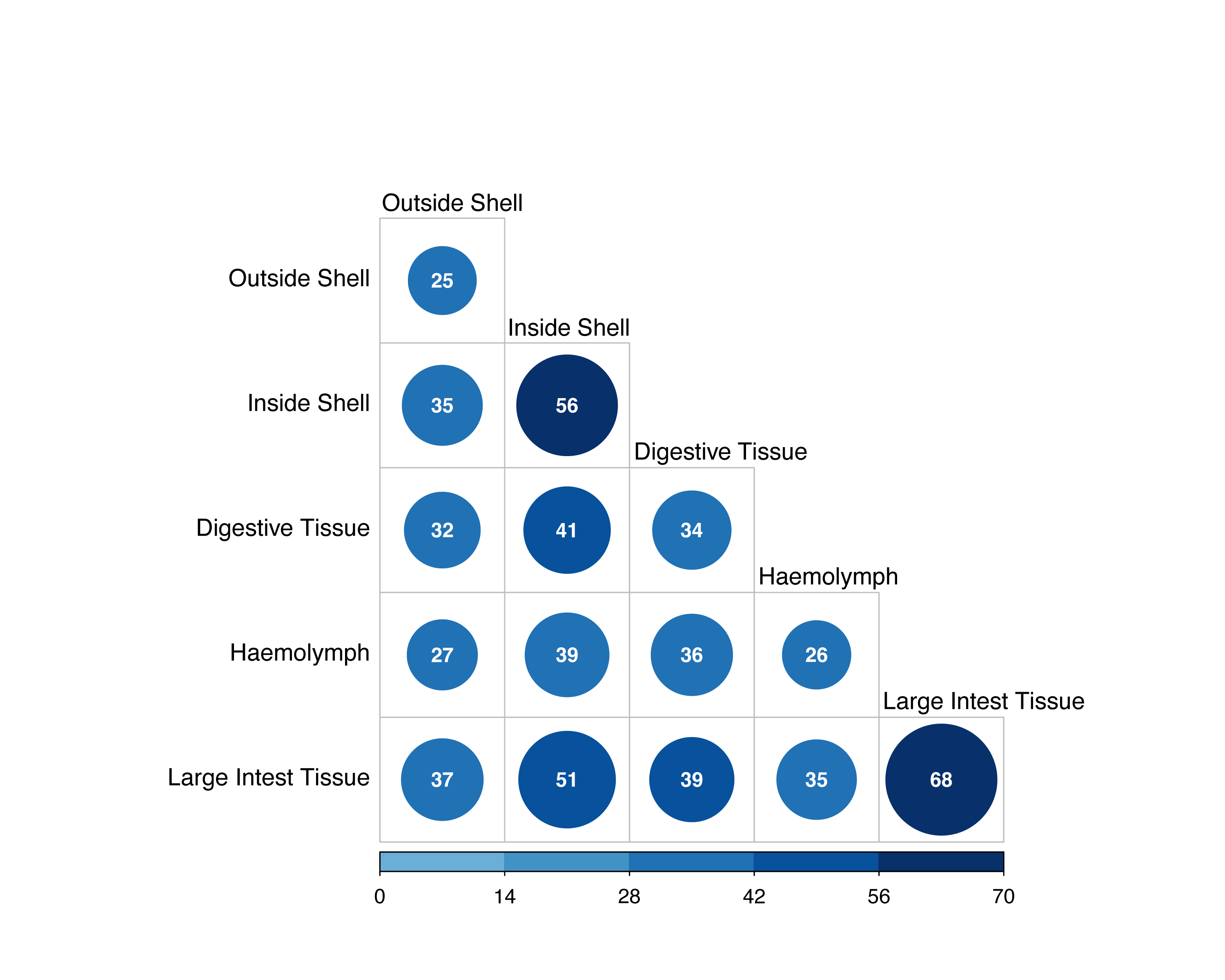

Supplement: Supplementary Figure 2 — Heat map triangle showing average inter-group Bray-Curtis similarities of the Vibrio communities between tissue types. The circles show the within-tissue type similarities and larger circles and numbers represent greater similarities within and between tissue types. [file Image_2.TIFF]
